# Instruments to Assess Bidirectional Intimate Partner Violence: A Systematic Review

**DOI:** 10.1177/15248380251358230

**Published:** 2025-08-20

**Authors:** Olga Cunha, Marta Sousa, Sofia Faria, Ana Filipa Pinto, Andreia Machado

**Affiliations:** 1 Psychology Research Center (CIPsi), University of Minho, Braga, Portugal; 2 Hei-Lab: Digital Human-Environment Interaction Labs, Lusófona University, Porto, Portugal

**Keywords:** bidirectional violence, intimate partner violence, instruments, psychometric properties, prevalence, systematic review

## Abstract

Intimate partner violence (IPV) is a significant public health issue that affects individuals, families, and society in profound ways. Over the years, research has often shown that such IPV is predominantly one-sided, with men as perpetrators and women as victims. However, more recent studies have revealed that IPV is frequently bidirectional, with both partners potentially being victims, perpetrators, or both. Despite this growing awareness, little is known about the tools used to assess bidirectional violence (BV). This systematic review aims to synthesize the published scientific literature on the instruments used to assess bidirectional IPV among adult men and women. Following Preferred Reporting Items for Systematic Reviews and Meta-Analyses (PRISMA) guidelines, a search was conducted across databases such as PubMed, B-on, Web of Science, and Scielo. Fifty studies published between 2012 and 2024 met the inclusion criteria. Most of the studies were conducted with community samples recruited through online questionnaires. The Conflict Tactics Scale emerged as the most used instrument for evaluating BV, demonstrating robust psychometric properties. BV prevalence rates ranged from 2% to 98.4% and remained generally consistent across different instruments, except in the case of male reports. However, the limited number of studies on interpartner agreements and the reliance on single-partner self-reports pose challenges to the accuracy of these estimates. While this systematic review provides a comprehensive overview of the instruments used to assess BV, it underscores the need for future research to develop more precise, context-sensitive tools, that incorporate reports from both partners to improve the accuracy of BV assessment.

Intimate partner violence (IPV) encompasses abusive or coercive behaviors by one partner to control, intimidate, or cause harm to the other (Laskey et al., 2019; Miller & McCaw, 2019), including physical aggression, sexual coercion, psychological abuse, and controlling behaviors (World Health Organization [WHO], 2022). It represents a significant public health issue, affecting millions each year (Sardinha et al., 2022) and leading to serious consequences for victims and their families (Centers for Disease Control and Prevention, 2022; Machado & Farinha, 2023; Stubbs & Szoeke, 2022).

This phenomenon has long been observed from a patriarchal and unidirectional perspective, in which men control and exert power over women (Bates, 2016; Langhinrichsen-Rohling et al., 2012; Laskey et al., 2019; Lewis et al., 2015). This form of violence is generally associated with higher levels of dominance and control by one partner, particularly in cases of gender-based violence, such as male-perpetrated violence against women (E. Miller & McCaw, 2019; Umoren & Owiriwa, 2018). However, several typologies of violence have emerged, indicating the existence of other patterns of violence than unidirectional ones (Bates, 2016). Johnson’s (2006) typology categorizes IPV into four main types, highlighting how power dynamics and motivations can differ. Intimate Terrorism (IT) involves one partner exerting control over the other through persistent abuse, often creating a significant power imbalance, with one partner acting as the primary perpetrator. Situational Couple Violence (SCV) arises from conflicts that escalate into violence but generally lack an overarching intent to control the partner. SCV is generally less severe and may be more likely to involve both partners in conflict. In Violent Resistance (VR), one partner responds to the aggression or control of another with violence, often as an act of self-defense. Finally, Mutual Violent Control (MVC) occurs when both partners use violence to control each other. Johnson (2006) also notes that IPV may be unidirectional, where only one partner is violent, or bidirectional, where both partners are involved in aggression. Therefore, it is unanimous that men and women can be victims and/or perpetrators (Machado et al., 2019, 2024). Unidirectional and bidirectional violence (BV) represent different phenomena in terms of their dynamics and consequences (Palmetto et al., 2013; Ridings et al., 2018).

## Bidirectional IPV

BV occurs when both partners engage in violent behavior, either simultaneously or alternately. This form of violence involves both partners using violence, which adds complexity to the situation. Such reciprocal violence can be seen in both heterosexual and homosexual relationships (Bates, 2016; Johnson, 1995, 2006; Ridings et al., 2018).

Several studies identify BV as the most prevalent form of IPV (e.g., Langhinrichsen-Rohling et al., 2012; Machado et al., 2024). In Europe, a survey conducted across six cities found BV to be the most common type, with 10% of women and 11.9% of men reporting experiences as either victims or perpetrators of physical violence. Moreover, women report experiencing lower severity acts more frequently than men (22.1% vs. 12.5%; Costa et al., 2016). Studies conducted among male and female samples (e.g., Capinha et al., 2022; Hines & Douglas, 2018, 2019; Machado et al., 2019), college students (e.g., Juan & Madan, 2018; Santiago et al., 2025), IPV survivors (e.g.,Holmes et al., 2016), and heterosexual and non-heterosexual samples (e.g., Capinha et al., 2022) also found that BV is the most prevalent pattern of violence, with psychological violence being the most perpetrated and suffered. Furthermore, evidence has shown that, in some couples, BV may be as common or more common than unidirectional violence (Langhinrichsen-Rohling et al., 2012; Straus, 2015). A systematic review by Langhinrichsen-Rohling et al. (2012) confirmed these findings, with BV appearing in 57.5% of intimate relationships, making it the most frequently observed pattern across all samples. More recently, a systematic review by Machado et al. (2024) found prevalence rates of BV from 2% to 98.4% and that this pattern of violence was the most reported across different countries and samples.

However, as many studies fail to consider the context and intent behind violent acts, comparing the frequency of violence can lead to couples being perceived as having BV, even when one partner is merely acting in self-defense (Babcock et al., 2019). Failing to account for the context of violence or the motivations behind its use can also influence how victims and perpetrators label themselves as victim, perpetrator, or victim-perpetrator. For instance, it is known that perpetrators of IPV often attribute their violent behavior to uncontrollable factors, such as their partner’s use of violence against them (Cunha et al., 2022). In fact, the motives for BV can vary from self-protection to attempts at retaliation or control of the situation (Langhinrichsen-Rohling et al., 2012).

Alongside its high prevalence, BV has serious implications for victims and society. On a personal level, this type of IPV can potentially lead to more severe psychological and physical harm compared to unidirectional violence, and there is a greater likelihood of escalation and increased severity (Fernández-Montalvo et al., 2020; Holmes et al., 2016). Prolonged exposure to such environments can lead to psychological problems, including depression, anxiety, and post-traumatic stress disorder, along with physical injuries that range from minor harm to severe trauma (Hellmuth et al., 2014; Jennings et al., 2012; Ulloa & Hammett, 2014). From a public health perspective, BV violence represents a substantial challenge, as it requires interventions that address both perpetrators and victims, recognizing that in many cases the roles are interchangeable. The public health impact goes beyond the immediate treatment of victims and perpetrators, as this form of violence can perpetuate cycles of intergenerational abuse, affecting children who grow up in violent homes and who may replicate these abusive behaviors in their own adult relationships (Cui et al., 2013; Rakovec-Felser, 2014).

BV is often associated with a vicious cycle of aggression, where partners fuel hostile behaviors. Deficits in conflict resolution skills also characterize BV, as many couples who engage in violent behavior have difficulties in dealing with conflicts constructively, resorting to violence as a way of coping with frustrations and tensions in the relationship (Hine et al., 2022; Neilson et al., 2023). Another characteristic is the cyclical nature of violence, where BV can be perpetuated by a cycle of provocation and response, where one violent behavior triggers another, leading to an escalation of aggression (Palmetto et al., 2013; Ridings et al., 2018). Both partners contribute to maintaining this cycle, making the violence reciprocal (Holmes et al., 2016). Situational and emotional factors, such as emotional stress, financial difficulties, mental health problems, might also be important factors (Palmetto et al., 2013; Ridings et al., 2018).

## Measuring Bidirectional IPV

Interpersonal violence is recognized as a sensitive and complex research topic, introducing significant challenges in terms of their measurement as most of this violence is not possible to measure objectively without asking those involved (Fraga, 2016). Data on violence experiences and IPV in particular depends on several factors, such as the type of instrument used, how the questions are asked, and the modes of questionnaire administration (Costa & Barros, 2016; Fraga, 2016). For example, researchers seem to agree that direct questioning about experiences of specific acts of violence over a particular period, the so-called self-report measures, should be used rather than using more open-ended and generic questions (Costa & Barros, 2016).

Indeed, research on IPV frequently depends on self-report instruments to gather data (Costa & Barros, 2016; Woodin et al., 2013). Nonetheless, numerous studies have underscored the methodological limitations inherent in this approach (Gibbs et al., 2019). For instance, cognitive interviewing studies—a method where participants verbalize their thought process while answering survey items—revealed discrepancies between how individuals interpret and respond to questions and the researchers’ expectations (Willis, 2004). Since self-reports are grounded in individuals’ personal accounts of events, what is actually being measured are subjective narratives about violence, rather than the violent acts themselves. Moreover, self-report data are susceptible to recall bias; participants may misremember the timing of incidents or choose to misreport information due to legal concerns or the desire to present themselves in a socially acceptable light (Hamby, 2014; O’Sullivan, 2008). In addition, self-report instruments do not seem to represent “most data” on IPV as when these sources are considered and placed in the context of other data on violence and aggression, a clear pattern of gender asymmetry emerges, with males perpetrating more physical and sexual violence than females for virtually every form of violence ever studied (Hamby, 2014). Moreover, the stability of self-reported measures of IPV remains a significant concern (Gibbs et al., 2019).

The Conflict Tactics Scale (CTS), both the original or modified and revised versions (Straus, 1979; Straus et al., 1996; Straus et al., 2003), is arguably the most widely accepted and frequently used self-report instrument in the field of IPV (e.g., Costa & Barros, 2016). A recent systematic review also found that CTS was the instrument most commonly used to assess bidirectional IPV (Machado et al., 2024). This tool includes 78 self-report items across several subscales, assessing the frequency and severity of aggression both perpetrated and experienced by each partner. CTS has been considered an innovative tool due to their emphasis on behavioral descriptions, avoiding terms such as “abuse” that are still sometimes used in survey research despite extensive data documenting the problems with such language (Hamby, 2014).

Although widely used, CTS has notable shortcomings. One key criticism is its inability to capture gender-specific dynamics in IPV, such as who initiates the violence, the underlying motivations, and the differential psychological and emotional impacts experienced by each gender (Dokkedahl et al., 2019; Langhinrichsen-Rohling et al., 2012). Moreover, the instrument has been described as overly reductionist, focusing narrowly on whether a violent act occurred while largely overlooking the broader context in which that act took place (Chapman & Gillespie, 2019; Dokkedahl et al., 2019). Contemporary research increasingly emphasizes that identifying IPV accurately requires more than a checklist approach; it demands attention to the situational and relational contexts surrounding violent interactions. Furthermore, critics argue that CTS may misclassify certain behaviors and individuals, potentially overestimating the frequency and severity of women’s IPV in some samples (Lehrner & Allen, 2014). For instance, studies comparing CTS-based classifications with findings from clinical interviews and audiotaped scenarios have shown limited agreement, with the CTS often producing higher prevalence estimates of both victimization and perpetration (Lehrner & Allen, 2014).

When assessing bidirectional IPV, the limitations of self-report measures become even more evident. Although IPV is inherently relational and numerous studies report high rates of BV (see Machado et al., 2024), most research still depends on the perspective of a single partner (Armstrong et al., 2002). This one-sided approach can introduce significant biases, raising concerns about the validity of the information gathered when only one member of the dyad provides accounts of violent interactions (Capinha et al., 2024). Evidence suggests that findings may vary substantially depending on whether the reports come from men or women (Dobash & Dobash, 2004; Follingstad & Rogers, 2013; Hamby, 2014), and that responses are also shaped by the respondent’s relationship to the perpetrator and the broader social context (Felson & Paré, 2005). Importantly, such differences in reporting are not always due to variations in the actual events but may instead reflect discrepancies in how those events are perceived, remembered, and described by victims and perpetrators (Dobash & Dobash, 2004). This issue is particularly relevant in the context of bidirectional IPV. Although the developers of the CTS claimed that consistent results were obtained from male and female respondents (Straus et al., 1996), subsequent research has found only modest levels of agreement between partners regarding both the occurrence and frequency of IPV.

Overall, these methodological challenges, along with the absence of assessment tools designed specifically for bidirectional IPV, pose significant obstacles to fully understanding this complex pattern of violence. They also undermine the development of effective, evidence-based interventions and prevention strategies.

## Current Study

Although research on IPV has traditionally focused on unidirectional patterns, portraying men as the primary perpetrators and women as the primary victims, there is growing interest in understanding bidirectional IPV, where both partners can be victims, perpetrators, or both. However, considerable debate surrounds the assessment of BV, and little is known about the instruments used to measure it.

This systematic review aims to synthesize published scientific literature on instruments used to assess bidirectional IPV, with a specific focus on dyadic interactions within intimate relationships—capturing both victimization and perpetration by both partners among adult men and women. Specifically, it seeks to: (a) identify the most commonly used instruments for assessing bidirectional IPV; (b) review the psychometric properties of these instruments; (c) examine variations in instruments based on sample type (i.e., clinical, forensic, community); (d) explore differences in instruments based on data collection methods (i.e., self-administered, face-to-face, telephone); and (e) assess how prevalence rates of bidirectional IPV vary across different instruments.

## Method

This systematic literature review adhered to PRISMA (Preferred Reporting Items for Systematic Reviews and Meta-Analyses) guidelines (Page et al., 2021) and utilized the following databases—PubMed, B-on, Web of Science, and Scielo—selected for their relevance to interdisciplinary research in psychology, criminology, and public health, which are key domains in the study bidirectional IPV (and unidirectional IPV). The equation search was the following: (“intimate partner violence” OR “domestic violence” OR “mutual violence” OR “bidirectional* violence” OR “symmetrical violence” OR “perpetrator-victim role” OR “situational partner violence” OR “victim-perpetrator overlap” OR “couple violence” OR “reciprocal violence” OR “mutual control”) AND (“instrument” OR “tool” OR “scale” OR “questionnaire” OR “inventory”) OR (“incidence” OR “prevalence” OR “rate”), which was run by two reviewers in July 2024. The search terms were chosen based on previous literature, with the aim of capturing bidirectional IPV within dyadic interactions in intimate relationships. Following an initial screening of titles and abstracts, full texts not accessible online were requested directly from the corresponding authors. Articles were excluded if the authors did not respond. Additional studies were incorporated based on a backward search of the references cited in the selected studies. Lastly, a basic Google search was performed to approximate the verification of the search strategy. The systematic review was registered on the OSF REGISTRIES (reference: https://doi.org/10.17605/OSF.IO/ERHZQ.

### Inclusion/Exclusion Criteria

To be included in the present systematic review, articles should be (a) based on empirical data (quantitative, qualitative, or mixed methods) and report bidirectional IPV prevalence; (b) evaluate measurement-related issues concerning bidirectional IPV; (c) written in Portuguese, Spanish, or English; (d) include adult women and/or men participants; and (e) published in a peer-reviewed journal. No restrictions regarding the year of publication were made. For exclusion, the criteria included studies that involved juvenile samples, as adult relationships differ from adolescent and juvenile ones in terms of developmental stages, contextual and legal frameworks, violent dynamics, and measures used to assess them. Additionally, review papers (i.e., scoping and systematic reviews) and grey literature (e.g., dissertations, theses, conference papers) were excluded.

### Literature Selection Process and Data Extraction

The selected studies were imported into Rayyan software (Ouzzani et al., 2016) to remove duplicates. Two authors independently screened titles and abstracts for inclusion. After that, the two reviewers independently extracted data from the included studies using a codebook to gather information on the following topics: study information (e.g., year of publication, country, design), sample information (e.g., gender, sample size, mean age), measurement’s characteristics (e.g., assessment measures; psychometric properties), and findings. Discrepancies between the two raters in these two phases were reviewed and discussed, with a third reviewer available to intervene if needed.

### Quality Assessment

To assess the methodological quality of all included studies, the Quality Assessment Tool for Studies with Diverse Designs (QATSDD) was utilized (Sirriyeh et al., 2012). The QATSDD is a 16-item instrument designed to assess methodological quality, evidence quality, and quality of reporting across qualitative, quantitative, mixed- and multi-methods studies. Each item is scored from 0 to 3, with higher scores indicating greater methodological rigor. For qualitative and quantitative studies, total scores on the QATSDD can range from 0 to 42, and up to 48 for mixed-method studies. To ensure accuracy, the QATSDD was designed to provide an overall quality rating expressed as a percentage. The QATSDD assigns a percentage score to assess and compare the quality of reporting across studies. To simplify comparisons, studies are categorized as low quality (≤50%), moderate quality (51%–74%), or high quality (≥75%) (Crowe et al., 2011).

### Data Analysis

The methodology followed a narrative synthesis approach (e.g., Popay et al., 2006), using descriptions and tables to summarize the data. This approach enables readers to assess the findings while accounting for variations in study designs, sample types, settings, and potential biases present in the reviewed studies. Moreover, chi-squared tests were used to examine associations between nominal variables, such as the data collection method (i.e., online or face-to-face) and the collection context (i.e., community, clinical, or forensic settings), and the assessment instruments employed. ANOVA tests were conducted to examine differences in assessment instruments based on the prevalence of BV. The variables for these analyses were created based on the data extracted from the manuscripts included in the systematic review.

## Results

### Screening and Selection of Studies

**Figure 1.**
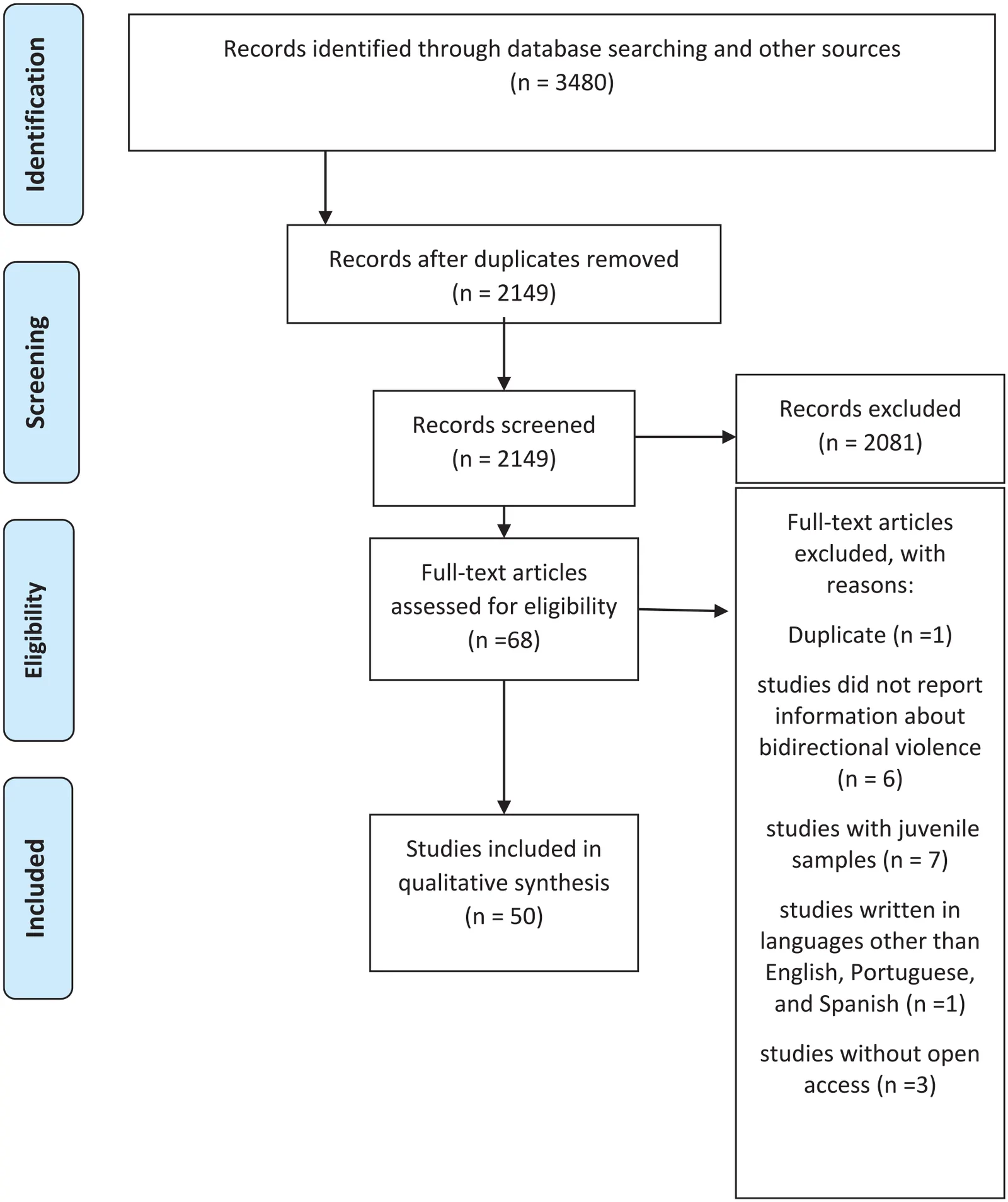
PRISMA flow diagram of the study selection process.

A total of 3,440 papers were retrieved from database searches and 40 from supplementary searches. After removing duplicates, 2,149 titles and abstracts were screened for relevance. Sixty-eight studies were selected for further examination. One was found to be duplicate, and 17 further studies did not meet the inclusion criteria. The main reasons for exclusion were: studies did not report information about BV, studies with juvenile samples, studies written in languages other than English, Portuguese, and Spanish, and studies without full text available. Fifty studies were included in the final review. Figure 1 shows the PRISMA flow diagram illustrating the number of included studies in each selection process and the reasons for the studies’ exclusion.

### Quality Assessment

The current systematic review included articles with acceptable quality (*n* = 35) and high quality (*n* = 15). The studies by Hines and Douglas (2018, 2019) achieved the highest quality score at 93%, followed by five studies with quality scores in the 80% range (Bryan et al., 2019; Chen & Chan, 2021; Machado et al., 2019; Muftić et al., 2015; Stults et al., 2020). Thirteen studies achieved a total score in the 70% range (Arteaga et al., 2015; Brown & Chew, 2018; Caetano et al., 2024; Capinha et al., 2022; Conroy et al., 2022; Frías, 2017; Hu et al., 2019; Leiding et al., 2022; Norris et al., 2017; Sommer et al., 2016; Stephenson et al., 2020; Tillyer & Wright, 2013; Trillingsgaard et al., 2019), followed by 22 studies with quality scores in the 60% range (see Tables 1 and 2). Only eight studies are classified as acceptable with a quality score in the 50% range (Costa et al., 2018; Frankland & Brown, 2014; Juan & Madan, 2018; Lewis et al., 2015; Malik & Nadda, 2019; McKeown, 2014; Seewald et al., 2022; Suomi et al., 2018).

**Table 1.** Sample Characteristics of the Included Studies.

Author, Year	Country or Continent, Setting Recruited Population	Sample Size, Gender	Sample Mean Age (SD)
Afifi et al., 2012	USA, community	25,778, both	N/A
Arteaga et al., 2015	Spain, health care	162, both	36.40 (8.9)
Behnken et al., 2018	USA, community	763, women	N/A
Brown and Chew, 2018	Singapore, forensic	150, both	N/A
Bryan et al., 2019	USA, healthcare clinics	77, male	43.7 (12.7)
Caetano et al., 2024	USA, healthcare clinics	1,037, both	35.2
Capinha et al., 2022	Portugal, community	1,392, both	Male: 42.67 (13.17) Female: 32.74 (11.79)
Carranza et al., 2020	Canada, community	1,018, both	19.84 (2.95)
Chen and Chan, 2021	China, community	7,466, both	40.95 (8.66)
Conroy et al., 2022	Malawi, clinical	211 couples, both	40.5
Costa et al., 2014	Europe, community	3,496, both	N/A
Costa et al., 2015	Europe, community	3,496, both	N/A
Costa et al., 2016	Europe, community	3,496, both	N/A
Costa et al., 2018	Europe, community	3,279, both	N/A
Crane et al., 2014	USA, forensic	68, male	39.13
Cunha et al., 2024	Portugal, community	336, both	35.02 (11.67)
Flake et al., 2013	Brazil, community	362, both	N/A
Fernández-Montalvo et al., 2020	Spain, clinical	122, both	36.6
Frankland & Brown, 2014	Australia, community	184, both	N/A
Flanagan et al., 2014	USA, clinical	180, women	N/A
Frías, 2017	Mexico, community	114,242, women	N/A
Hester et al., 2015	UK, healthcare clinics	707, male	43 (15)
Hines and Douglas, 2018	USA, forensic and community	2,212, male	41.77 (22.35) 43.89 (9.18)
Hines and Douglas, 2019	USA, forensic and community	1,112, male	Community sample: 41.77 (11.35) Forensic sample: 43.89 (9.18)
Holmes et al., 2016	USA, forensic	227, women	35
Hu et al., 2019	China, community	1,301, male	27.2
Juan and Madan, 2018	Mexico, community	597, both	N/A
Kubicek et al., 2015	California, community	101, male	21.5
Leiding et al., 2022	Germany, healthcare clinics	4,601, male	Victims and perpetrators: 47.9 (17.8)
Lewis et al., 2015	USA, community	414, women	29.26
Machado et al., 2019	Portugal, community	1,556, male	32.58
Malik & Nadda, 2019	India, community	1,000, male	N/A
Marcus, 2012	USA, community	1,294 couples, both	22.3
McKeown, 2014	UK, forensic	92, women	N/A
A. P. Miller et al., 2022	Uganda, healthcare clinics	408, both	N/A
Muftić et al., 2015	USA, forensic	1,256, Both	32.9 (9.7)
Norris et al., 2017	Africa, community	158, male	N/A
Renner and Whitney, 2012	USA, community	10,187, both	21.80
Richards et al., 2017	USA, community	14,287, both	Male: 28.51 Female: 28.31
Seewald et al., 2022	USA, community	1,079, male	26.5
Sommer et al., 2016	USA, community	258 couples, both	Male: 31.37 Female: 29.32
Stephenson et al., 2020	South Africa and Namibia, community	440, male	N/A
Stults et al., 2020	New York, community	26, male	26.4
Suomi et al., 2018	Australia, clinical	212, women	43.6 (13.0)
Tillyer & Wright, 2013	USA, community	4,275, both	28.43
Trillingsgaard et al., 2019	Denmark, clinical	1,726 couples, both	Male: 31.2 Female: 29.4
Ulloa & Hammet, 2014	USA, community	7,187, both	Male: 22.38 Female: 29.12
Vasconcelos et al., 2023	Portugal, community	138, both	38.98 (7.34)
Wei et al., 2020	China, community	578, male	N/A
Wu et al., 2015	New York, community	74 (37 couples), male	41.8

**Table 2.** Main Findings.

Author, Year	BV Measures; Psychometric Properties	Data Collection	Prevalence of BV	Quality Percentage and Category
Afifi et al., 2012	CTS; N/A	Face to face interviews	M: 3.1% F: 3.9%	62%, acceptable
Arteaga et al., 2015	CTS-2; N/A	Self-administered Face to face (paper-and-pencil)	Overall: 98.4%	79%, high
Behnken et al., 2018	CTS-2; N/A	Face to face interviews	Overall: 41% PA: 68.7%–79.6% SC: 32.1%–46.5% PV: 59.4%–71.2% I: 7.7%–12.5%	62%, acceptable
Brown and Chew, 2018	CTS-2; α = .79–.95	Self-administered Face to face (paper-and-pencil)	M - MVC: 17%–20% F – MVC: 14.8%–15.8%	79%, high
Bryan et al., 2019	CTS-2; N/A	Self-administered Face to face	PV: 69.6% PA: 57.9% CS: 35.3%	86%, high
Caetano et al., 2024	CTS-2 PA scale; α = .85	Face to face interviews	Overall: 13.3% M: 13.4% F: 13.3%	76%, high
Capinha et al., 2022	CTS-2; α = .72–0.95; SC victimization and perpetration: α < .50; PV: .64	Self-administered Web-based survey	Hetero: 89.1% Non-hetero: 84.1%	74%, acceptable
Carranza et al., 2020	CTS-2; PA perpetration: α = .95; PA victimization: α = .95; PV perpetration: α = .79; PV victimization: α = .79; SC perpetration: α = .83); SC victimization: α = .83	Self-administered Web-based survey	Overall: 66.7%	62%, acceptable
Chen and Chan, 2021	CTS-2; α = .77–.87	Self-administered Face to face	PA: 61.13% PV: 80.58% I: 47.58% SC: 38.53%	83%, high
Conroy et al., 2022	A questionnaire with 12 questions for PA, 12 questions for SC, and 8 questions for SC; N/A	Face-to-face interviews Web-based survey	PA: 25.4% SC: 35.5% PV: 34.0%	71%, acceptable
Costa et al., 2014	CTS-2; Victimization: α = .90 (.83–.96); Perpetration: α = .90 (.75–.95)	Self-administered	Past-year PA: M: 11.9%; F: 10% Past-year SC: M: 12.5%; F: 9.7% Lifetime PA: M: 18.4%; F: 15.9% Lifetime SC: M: 11.4%; F: 7.5%	67%, acceptable
Costa et al., 2015	CTS2; N/A	Self-administered	PV: M: 54.5%; F: 54.4% SC: M: 12.5%; F: 9.7%	67%, acceptable
Costa et al., 2016	CTS-2 (PA scale); Victimization: α = .90 (.83–.96); Perpetration: α = .90 (.75–.95)	Self-administered	PA: M: 11.9%; F: 10%	69%, acceptable
Costa et al., 2018	CTS-2; N/A	Self-administered	Overall: 16.53% M:18% F: 15.5%	57%, acceptable
Crane et al., 2014	CTS-2; Baseline: α = .53–.83; Follow-up: α = .88–.92	Self-administered Face to face	PA: 54.54% I: 66.67% PV: 80% CS: 50%	62%, acceptable
Cunha et al., 2025	CTS2; α = .71 (total IPV victimization) to .96 (total IPV perpetration)	Self-administered Web-based survey	Overall: 31.3%	67%, acceptable
Flake et al., 2013	CTS-2; N/A	Self-administered Face to face	Overall: 83.9%	69%, acceptable
Fernández-Montalvo et al., 2020	CTS-2; α = .946	Face to face interview	Overall: 32% M: 56.1% F: 83.9%	64%, acceptable
Frankland & Brown, 2014	CTS-2; Perpetration - PV: α = .79; PA: α = .90; CS: α = .84; I: α = .76; Victimization - PV: α = .76; PA: α = .91; CS: α = .77; I: α = .76	Self-administered Web-based survey	Overall: 12.5 %	52%, acceptable
Flanagan et al., 2014	CTS-2; Perpetration - CS: α = .77; PV: α = .74; PA: α = .91; Victimization - CS: α = .72; PV: α = .84; PA: α = .92	Self-administered Face to face	Baseline: 7.2% Follow-up: 5.7%	60%, acceptable
Frías, 2017	CTS; NA Questions about perpetration and victimization behavior; N/A 11 items assessing CB: α = .82 (married women) and .86 (separated women)	N/A	26.7% (married and cohabiting) 29.3% (separated and divorced)	71%, acceptable
Hester et al., 2015	4 questions about IPV behaviors; N/A COHSAR adapted; N/A	Self-administered Face to face	Overall: 8.6%	60%, acceptable
Hines and Douglas, 2018	CTS2; α = .69 (severe PV perpetration) to .94 (PA victimization) 9 items of the PMWI; N/A 12 items assessing legal/administrative aggression; N/A	Self-administered Web-based survey	MSCV - Total sample: 10.4% Population-based sample: 10.0% Male victims: 11.4% MVC – Total sample: 2.8%; Population-based sample: 2.7%; Male victims: 3.2	93%, high
Hines and Douglas, 2019	CTS2 (PA scale); PA perpetration: α = .90 PA victimization: α = .94 9 items of the PMWI; N/A CB perpetration: α = .84; CB victimization: α = .89	Self-administered Web-based survey	MSCV – Total sample: 10.4%; Population-based sample: 10.0%; Male victims: 11.4% MVC – Total sample: 2.8%; Population-based sample: 2.7%; Male victims: 3.2%	93%, high
Holmes et al., 2016	CTS2; α = 69–.79	Face-to-face interviews	Overall: 5.3% PA: 3.5% SC: 1.3% PV: 0.4%	67%, acceptable
Hu et al., 2019	CTS2 (adapted); α = .76–.90	Self-administered Web-based survey	All types: 3.3% PV: 34.6% All types minus PA: 26.3%	79%, high
Juan and Madan, 2018	14 questions developed for the study; N/A	N/A	M: 60.0% F: 53.9%	57%, acceptable
Kubicek et al., 2015	Semi-structured Interview CTS2; N/A	Computer-assisted Self-administered	PV: 10%–69% PA: 3%–38% SC: 3%–28% I: 4%–18%	60%, acceptable
Leiding et al., 2022	52 questions to assess IPV victimization and perpetration; N/A Violence-related questions to assess PA, PV, CS; N/A	Self-administered Face to face	25%	74%, acceptable
Lewis et al., 2015	CTS2 (PA scale), N/A	Self-administered Web-based survey	Overall: 12%	55%, acceptable
Machado et al., 2019	CTS2; SC: α = .76; PV: α = .87; PA: α = .93; I: α = .95	Self-administered Web-based survey	73.7%	88%, high
Malik & Nadda, 2019	NFHS-3; α = .86	Face to face interviews	PA = 46%	52%, acceptable
Marcus, 2012	Questionnaire to assess the frequency of perpetration and victimization; N/A	Face-to-face interviews	Overall: 10% (couples reports); 18% (single partner reports)	60%, acceptable
McKeown, 2014	CTS-2; N/A	Self-administered Face to face	Current relationship - PA: 56%; PV: 88% Previous relationship - PA: 86%; PV: 93%	50%, acceptable
A. P. Miller et al., 2022	CTS-2 (adapted); N/A	Face to face interviews	Past 6 months – Overall: 2.7%; F: 1.5%; M: 3.3% Lifetime – Overall: 8.1%; F: 7.6%; M: 3.3%	69%, acceptable
Muftić et al., 2015	Categorization according to the police report (victim, offender, or victim-offender), N/A	Police records	18.8%	86%, high
Norris et al., 2017	CTS2 PA scale (adapted); N/A	Computer-assisted Self-administered	PA: 11%	76%, high
Renner and Whitney, 2012	4-item questionnaire to assess IPV perpetration and victimization; N/A	Face to face interviews	Overall: 25.4% M: 23.27% F: 27.36%	64%, acceptable
Richards et al., 2017	CTS2 (adapted); N/A	Face to face interviews	M: 6.0% F: 7.3%	60%, acceptable
Seewald et al., 2022	5 TFA items, N/A	Self-administered Web-based survey	Overall: 25.6%	52%, acceptable
Sommer et al., 2016	CTS2; N/A	Telephone interview	Overall: 72.9%	76%, high
Stephenson et al., 2020	IPV-GBM; Victimization: α = .78; Perpetration: α = .76 Conflict Style Inventory; N/A	Computer-assisted Face-to-face interviews	Overall: 10.2%	74%, acceptable
Stults et al., 2020	Semi-structured Interview; N/A	Self-administered Face-to-face interview (semi-structured interview)	Overall: 65%	81%, high
Suomi et al., 2018	HITS Scale, N/A	Face to face interviews	Overall: 32.6%	55%, acceptable
Tillyer & Wright, 2013	Questions developed for this study; N/A	Self-administered	Overall: 6.7%	79%, high
Trillingsgaard et al., 2019	FMM; N/A	Self-administered Web-based survey	Low impact PA: 38.2% Clinically significant PA: 34.4% PV: 5.5%	79%, high
Ulloa & Hammet, 2014	CTS adapted; Wave 3 – Perpetration: α = .73; Victimization: α = .80; Wave 4 – Perpetration: α = .74; Victimization: α = .79	Face to face interviews	Wave 3: 12.30% Wave 4: 6.60%	62%, acceptable
Vasconcelos et al., 2023	IVC; Perpetration: α = .84; Victimization: α = .90	Self-administered Web-based survey	Overall: 35.9%	67%, acceptable
Wei et al., 2020	5 questions from the IPV-GBM; α = .71	Self-administered Web-based survey	PA: 6.60% PV: 11.80% CB: 4.5% MB: 9.3% SC: 4.7%	64%, acceptable
Wu et al., 2015	CTS2; α = .78–.93	Face-to face or telephone interviews	Overall: 62%	64%, acceptable

*Note.* N/A = Non available information; TFA = Technology-facilitated abuse; IPV-GBM Scale = Intimate Partner Violence Gay and Bisexual Men Scale; FMM = The Family Maltreatment Measure; MSCV = Mutual Situational Couple Violence; MVC = Mutual Violent Control; PA = Physical Aggression; PV = Psychological Violence; SC = Sexual Coercion; I = Injury; CB = Controlling behaviors; MB = Monitoring behaviors; IVC = Marital Violence Inventory; PMWI = Psychological Maltreatment of Women Inventory; NFHS-3 = National Family Health Survey-3; COHSAR = Comparing Heterosexual and Same Sex Abuse in Relationships.

### Characteristics of Included Studies

The year of publication of the papers varied between 2012 and 2024: 2012 (*n* = 3), 2013 (*n* = 2), 2014 (*n* = 6), 2015 (*n* = 7), 2016 (*n* = 3), 2017 (*n* = 3), 2018 (*n* = 6), 2019 (*n* = 7), 2020 (*n* = 4), 2021 (*n* = 1), 2022 (*n* = 5), 2023 (*n* = 2) and 2024 (*n* = 1). The empirical research included samples from various countries around the world, such as Portugal, Spain, Greece, Hungary, Sweden, Germany, Belgium, Brazil, the United Kingdom, Denmark, Malawi, Canada, South Africa, Namibia, Uganda, the USA, Mexico, China, Chile, Singapore, and Australia. Consequently, the samples spanned five continents: America (*n* = 25), Europe (*n* = 14), Asia (*n* = 5), Africa (*n* = 4), and Oceania (*n* = 2).

### Sample Characteristics and Methodology

The sample size of the studies ranged from 26 (Stults et al., 2020) to 114,242 individuals (Frías, 2017), with an average age ranging from 19.84 (Carranza et al., 2020) to 47.90 years (Leiding et al., 2022). However, almost half of the studies did not report the mean age of the participants. Twenty-seven were composed of both men and women, 16 samples were entirely composed of male individuals, and seven studies included only female participants (see Table 1). Only five studies included couples (e.g., Conroy et al., 2022; Marcus, 2012; Sommer et al., 2016; Trillingsgaard et al., 2019; Wu et al., 2015). Thirty-two studies recruited their participants in the community, 11 in healthcare clinics, and five in forensic settings. Two studies used both forensic and community samples.

Fourteen of the studies were conducted through an online questionnaire, three were computer-assisted (Kubicek et al., 2015; Norris et al., 2017; Stephenson et al., 2020), one used police reports (Muftić et al., 2015), one was conducted by telephone (Sommer et al., 2016), one employed a mixed methodology (i.e., face-to-face or telephone; Wu et al., 2015), and the remaining studies used questionnaires or interviews administered in person. Two studies did not report this information (Frías, 2017; Juan & Madan, 2018).

### Assessment Instruments Used

Most studies (*n* = 34) assessed BV using the CTS, or the revised/modified versions of CTS (Straus et al., 1996), or some items of CTS. However, one study relied on police reports to classify the types of violence experienced and perpetrated (Muftić et al., 2015), while others (*n* = 7; Conroy et al., 2022; Juan & Madan, 2018; Leiding et al., 2022; Marcus, 2012; Norris et al., 2017; Renner & Whitney, 2012; Seewald et al., 2022) utilized a series of questions derived from IPV definitions. Eight studies used other tools designed to assess IPV, such as the Family Maltreatment Measure (FMM; Trillingsgaard et al., 2019), the Intimate Partner Violence Gay and Bisexual Men Scale (IPV-GBM; Stephenson et al., 2020; Wei et al., 2020), the Marital Violence Inventory (IVC; Vasconcelos et al., 2023), the Psychological Maltreatment of Women Inventory (PMWI; Hines & Douglas, 2018, 2019), the National Family Health Survey-3 (NFHS-3; Malik & Nadda, 2019), and the Hurt-Insult-Threaten-Scream (HITS; Suomi et al., 2018). One of the studies that used the CTS included additional screening questions (Frías, 2017). The qualitative empirical study gathered data through a semi-structured interview (Stults et al., 2020), while the mixed-methods study employed both a semi-structured interview and the CTS (Kubicek et al., 2015). Importantly, some studies used more than one questionnaire to assess bidirectional IPV (e.g., Hines & Douglas, 2018, 2019).

### Measurement/Psychometric Properties of the Assessment Instruments Used

One commonly used tool in the included studies is the CTS (both the original or modified version and revised versions; Straus, 1979; Straus et al., 1996, 2003), which measures an individual’s involvement in or exposure to physical or psychological violence within an intimate relationship. CTS is a self-report measure assessing different types of violence and negotiation tactics. The instrument evaluates both the frequency and intensity of aggression experienced or perpetrated by each partner. Concerning the psychometric properties of the CTS, 18 studies reported Cronbach’s alpha coefficients for the instrument, which range from .53 (Crane et al., 2014) to .96 (Cunha et al., 2024). Specifically, the Cronbach’s alpha coefficients for the CTS physical aggression subscale ranged from .85 (Caetano et al., 2024) to .96 (Costa et al., 2014, 2016), for the psychological violence scale ranged from .85 (Caetano et al., 2024) to .95 (Carranza et al., 2020), and for sexual coercion ranged from <.50 (Capinha et al., 2022) to .83 (Carranza et al., 2020). In most studies, Cronbach’s alpha coefficients for the CTS, both for total scales (victimization and perpetration) and subscales, were higher than .70, with only four studies revealing values lower than the acceptable value of .70 (Capinha et al., 2022; Crane et al., 2014; Hines & Douglas, 2018; Holmes et al., 2016).

Another instrument used in two included studies (Stephenson et al., 2020; Wei et al., 2020) was the IPV-GBM Scale. The IPV-GBM assessed both victimization and perpetration of IPV in the past 12 months, adapted from the CTS to better capture IPV among gay and bisexual men (Stephenson & Finneran, 2013). The scale includes 23 items across four IPV domains: physical/sexual, emotional, controlling, and monitoring behaviors. Respondents were asked separately about their own experiences and their partner’s actions during the past year. The alpha of the instrument ranges from .71 (Wei et al., 2020) to .78 (victimization scale) (Stephenson et al., 2020)

Two other studies incorporated items from the PMWI (Hines & Douglas, 2018, 2019), which focuses on controlling behaviors. Additionally, one study used the FMM (Trillingsgaard et al., 2019), which includes questions based on the DSM-5 and ICD-11 criteria for clinically significant physical and psychological IPV, and another study used the HITS (Suomi et al., 2018). The HITS assesses four types of violence—physical harm, insults, threats, and yelling—perpetrated by partners or family members, both victimization and perpetration separately. However, none of these studies report Cronbach alphas or other reliability data of these measures.

One study assessed BV using the IVC (Vasconcelos et al., 2023). The IVC is a 21-item scale that measures both physical and psychological violence. It evaluates the prevalence of violence perpetrated and experienced by intimate partners. In this study, Cronbach’s alpha for the total scale was .84, and .90 for perpetration and victimization, respectively. Lastly, one study utilized the NFHS-3 (Malik & Nadda, 2019) to assess physical, sexual, and emotional violence. This questionnaire, adapted from the modified CTS, showed Cronbach’s alpha of .86.

### Assessment Instrument According to the Sample Type

CTS or its revised/modified versions were the most used instrument to assess the prevalence of BV in the different samples. Approximately two-thirds of the community-based studies (*n* = 22), half of the clinic-based studies (*n* = 6), and nearly all forensic-based studies (*n* = 4) use CTS or its revised/modified versions. Two studies were conducted with both community and forensic samples, using the CTS, the PMWI, and specific questions about IPV (Hines & Douglas, 2018, 2019).

Among clinical samples, two studies employed other validated measures—the HITS Scale (Suomi et al., 2018) and the FMM (Trillingsgaard et al., 2019). Additionally, two studies used a questionnaire with specific questions about violence (Conroy et al., 2022; Leiding et al., 2022), and one study employed a mixed-method approach combining screening questions and the Comparing Heterosexual and Same Sex Abuse in Relationships (Hester et al., 2015). Regarding forensic samples, only one study used a different measure from CTS and was based on police records (Muftić et al., 2015).

Among community samples, five studies used specific questions about IPV (Juan & Madan, 2018; Marcus, 2012; Renner & Whitney, 2012; Seewald et al., 2022; Tillyer & Wright, 2013), two used semi-structured interviews (Kubicek et al., 2015; Stults et al., 2020), two used the IPV-GBM (Stephenson et al., 2020; Wei et al., 2020), one used the IVC (Vasconcelos et al., 2023), and another used the NFHS (Malik & Nadda, 2019). The only study using a forensic sample that did not employ CTS relied on police records as its primary data source.

Chi-squared tests revealed no statistically significant associations between the instrument used to assess bidirectional IPV and the sample type, χ^2^ (9) = 6.370, *p* = .702.

### Assessment Instrument According to the Data Collection Method

Among the studies conducted through an online questionnaire, seven used CTS or its revised/modified versions, two used specific questions derived from IPV definitions (Conroy et al., 2022; Seewald et al., 2022), one used the FMM (Trillingsgaard et al., 2019), one used the IVC (Vasconcelos et al., 2023), and one used the IPV-GBM (Wei et al., 2020). One study used CTS and the PMWI (Hines & Douglas, 2019) and one study used the CTS, the PMWI, and specific questions to assess legal violence (Hines & Douglas, 2018).

Most studies conducted through face-to-face interviews used CTS (*n* = 8). The other studies used specific questions derived from IPV definitions (Marcus, 2012; Renner & Whitney, 2012), the NFHS (Malik & Nadda, 2019), and the HITS (Suomi et al., 2018). CTS were also used in the studies that collected data through telephone (Sommer et al., 2016) and telephone and face-to-face interview (Wu et al., 2015).

Studies collecting data through self-administered interviews used mostly CTS (*n* = 12), followed by specific questions derived from IPV definitions (Hester et al., 2015; Leiding et al., 2022; Tillyer & Wright, 2013). Computer-assisted studies used different measures to assess BV: CTS (Norris et al., 2017), IPV-GBM (Stephenson et al., 2020), and semi-structured interviews and CTS (Kubicek et al., 2015).

Chi-squared tests found no statistically significant associations between the data collection method and the instrument used to assess bidirectional IPV, χ^2^ (9) = 14.056, *p* = .120.

### Assessment Instrument According to the Prevalence of BV

The prevalence of BV in intimate relationships varied widely, ranging from 2% (in a study with a mixed clinical sample; A. P. Miller et al., 2022) to 98.4% (in a study with a mixed clinical sample of individuals undergoing treatment for substance dependence; Arteaga et al., 2015). The prevalence of physical violence ranged from 3% (Kubicek et al., 2015) to 86% (McKeown, 2014). The prevalence rate of psychological violence ranged from 0.4% (study conducted with a forensic sample of women shelter home residents; Holmes et al., 2016) to 93% (study conducted with women in a forensic setting; McKeown, 2014). Moreover, the prevalence of sexual coercion ranged from 1.3% (a study conducted with a forensic sample; Holmes et al., 2016) to 46.5% (a study conducted with a community sample; Behnken et al., 2018). Four studies reported a prevalence of injury, which ranged from 7.7% (Behnken et al., 2018) to 66.67% (Crane et al., 2014). Lastly, Wei et al. (2020) reported a prevalence of controlling behaviors of 4.5%.

ANOVA tests only revealed statistically significant differences in the instrument used to assess the prevalence of BV among male individuals, *F*(1,11) = 5.296, *p* = .042, ω = .325. Specifically, studies using questions derived from IPV definitions reported higher prevalence rates than those utilizing the CTS. ANOVA tests revealed no statistically significant differences in the prevalence of overall BV, *F*(3,47) = .192, *p* = .902, ω = .012, physical BV, *F*(3,20) = .155, *p* = .925, ω = .023, psychological BV, *F*(3,12) = 1.626, *p* = .235, ω = .289, sexual BV, *F*(3,15) = .588, *p* = .632, ω = .105, injury BV, *F*(1,4) = 1.089, *p* = .356, ω = .214, and BV among female individuals, *F*(1,7) = .942, *p* = .364, ω = .119.

Five studies assessed the concordance/agreement between partners: two studies used CTS (Sommer et al., 2016; Wu et al., 2015), two specific questions derived from IPV definitions (Conroy et al., 2022; Marcus, 2012), and one the FMM (Trillingsgaard et al., 2019). Most studies relied on the percentage/number of agreement/disagreement (Conroy et al., 2022; Trillingsgaard et al., 2019; Wu et al., 2015), while the other two used Kendall’s tau-b (Marcus, 2012) and Intraclass Correlations (Sommer et al., 2016). Conroy et al. (2022) found that 60.2% of couples agreed on physical IPV victimization and perpetration in their relationship. Discrepancies between partners were higher for sexual and emotional IPV. Trillingsgaard et al. (2019) reported a substantial percentage of agreement between partners (85.9%–88.7%), while Wu et al. (2015) found lower levels of interpartner agreement (73%–77%). Marcus (2012) and Sommer et al. (2016) also found high correlations between partners’ reports of IPV (.70 and .78, respectively). No significant associations were found between the instrument used and the percentage of agreement between partners, χ^2^ (8) = 10.000, *p* = .265, nor between the instrument used and the correlations of partners’ agreement in their reports, χ^2^ (1) = 2.000, *p* = .157.

## Discussion

This systematic review of BV in intimate relationships provides a comprehensive analysis of assessment instruments, prevalence, and sample characteristics in studies published to date. The findings reveal significant gaps in literature and highlight the inherent complexity of studying BV, suggesting the need for greater methodological and theoretical rigor. These results expand on the findings of Johnson et al. (2014), who argue that different forms of violence have distinct determinants, requiring tailored intervention approaches.

Our findings showed that publications on BV only began to emerge from 2012, marking the starting point for a notable increase in studies on this topic, which reflects the recent interest in exploring BV. The absence of studies until then may be linked to the patriarchal and unidirectional perspective that dominated the understanding of the IPV phenomenon, which primarily attributed violence to men to control and exert power over women (Bates, 2016; Langhinrichsen-Rohling et al., 2012; Laskey et al., 2019; Lewis et al., 2015). Half of the studies were conducted in America and nearly half used online methodologies for data collection. The collection of data through an online format presents certain limitations and could introduce some bias, particularly by restricting participants, such as older adults, individuals with lower levels of education, those with limited access to the Internet, or people who are less accustomed to using digital platforms and websites (Andrade, 2020; Capinha et al., 2024). Online surveys impede participants from clarifying any doubts, which may affect their response accuracy (Andrade, 2020; Nayak & Narayan, 2019). Additionally, online studies, often based on non-clinical samples, are more likely to identify minor intimate violence, as well as BV (Cunha et al., 2025). Most of the studies were based on community samples. However, forensic populations, such as perpetrators in the context of judicial intervention, or clinical populations, such as mental health patients, may present distinct patterns of violence. Further research is needed to explore the potential differences across countries in BV, using other samples and methodologies to collect data.

The results pertaining to assessment tools indicate that the CTS is the most used instrument for assessing BV, aligning not only with the existing literature in BV (e.g., Machado et al., 2024) but also with the literature conducted regarding unidirectional IPV (e.g., Costa & Barros, 2016). This is of extreme importance since the CTS has shown low negative correlations (or non-existent) with social desirability (Straus, 2004; Straus & Mickey, 2012). CTS includes less severe and conspicuous forms of psychological, physical, and sexual coercion, which may not have been accounted for in other assessment tools. This broader scope could improve the detection of these serious forms of maltreatment (Capinha et al., 2024). Besides, including specific behavioral questions—rather than relying on terms like “abuse”—appears to facilitate disclosure, as individuals are generally more likely to recognize the occurrence of a specific behavior than to label it as violence (Capinha et al., 2022; Hamby, 2014; Straus et al., 1996). However, a common critique of the CTS is its potential to misclassify certain behaviors and individuals, which may lead to an overestimation of the frequency and severity of women’s IPV in some samples (Lehrner & Allen, 2014).

Furthermore, in most studies using CTS, the psychometric properties of the instrument were acceptable or good, which is also consistent with prior research (e.g., Costa & Barros, 2016; Straus, 2004; Straus & Mickey, 2012). However, in four studies, the Cronbach’s alpha fell below the recommended threshold (>.70; cf. Field, 2017). Specifically, the sexual coercion subscale showed some lower values in comparison to the recommended (e.g., Capinha et al., 2022). Considering that the psychometric characteristics of a scale can be affected by a lack of variability in participants’ responses (Field, 2017), the low prevalence of reported sexual abuse found, suggesting a pattern of denial of such behaviors, may explain the lower values of internal consistency of this subscale. This does not necessarily indicate that sexual abuse is not prevalent, but rather, as the literature has reported, it is underreported (Wallis & Woodworth, 2020).

While the CTS exhibits robust psychometric properties, it has faced significant criticism (e.g., Babcock et al., 2019; Jones et al., 2017). A major limitation is its inability to account for gender-specific dynamics in IPV, the severity of injuries sustained, the context and motivations underlying violent behavior, or the consequences of such behavior (Dobash & Dobash, 2004; Dokkedahl et al., 2019; Jones et al. 2017; Langhinrichsen-Rohling et al., 2012). Like other self-report tools, the CTS does not capture information about who initiates the violence, making it difficult to distinguish between acts committed in self-defense or retaliation and those initiated without provocation. As a result, it overlooks the broader context in which the violent acts occur (Chapman & Gillespie, 2019; Dokkedahl et al., 2019). This limitation can lead to the misclassification of individuals who act in self-defense as engaged in BV (Babcock et al., 2019; Cunha et al., 2022), thereby complicating the interpretation of the directionality of IPV (Jones et al., 2017). Using CTS scores to assess BV (i.e., the presence or frequency of violent acts) risk overlooking critical contextual factors that influence the nature and consequences of such conflicts, thereby increasing the likelihood of identifying gender symmetry (Dobash & Dobash, 2004). Additionally, as a retrospective measure, the CTS depends on participants’ ability to accurately recall past events, which may be challenging for some individuals (Jones et al., 2017). Considering these limitations, Woodin et al. (2013) recommended supplementing self-report measures like the CTS with semi-structured clinical interviews. These interviews can provide critical insights into the context, motivations, impact, and frequency of violent acts, offering a more nuanced understanding of whether individuals are experiencing significant abuse.

The other standardized measures to assess BV (i.e., IVC; IPV-GBM) also revealed good psychometric properties, in accordance with prior research (Cunha & Gonçalves, 2016; Cunha et al., 2021; Stephenson & Finneran, 2013). Although less used, these instruments can capture important nuances in more vulnerable populations; however, due to their low utilization in the studies included in the current systematic review, no definitive conclusions can be drawn regarding their reliability. More extensive research is needed with these instruments.

A relatively small number of studies employed questions based on IPV definitions, rather than utilizing standardized measures, surveys, or instruments. This choice was likely driven by the researchers’ intent to provide a more targeted or detailed examination of the phenomenon, as existing tools may lack the sensitivity required to address their specific concerns. Notably, some of these studies were conducted within clinical samples using self-administered surveys, where the objectives extend beyond simply estimating BV prevalence. In such contexts, administering lengthy and structured questionnaires may not be practical (e.g., Costa & Barros, 2016). However, no statistically significant differences were observed in the type of questionnaire used to assess BV in relation to the sample or the data collection method.

In relation to the prevalence rates, our findings reported that BV ranged from 2% to 98.4%. This heterogeneity reflects the complexity of measuring BV, possibly due to methodological and contextual differences. For instance, studies that employed questions derived from specific definitions of violence often reported higher rates for men, suggesting that the operationalization of the BV concept can influence outcomes. Indeed, in a recent study, Zapata-Calvente et al. (2024) found that the detection of IPV improves when specific items are included, the IPV consequences are considered, and clear instructions are provided. Nonetheless, given the limited number of studies that utilized both specific questions and male samples, definitive conclusions cannot be drawn. Even so, employing reliable and standardized measures offers the advantage of better operationalizing the acts corresponding to each type and form of violence (Costa & Barros, 2016). For instance, the lack of a clear operational definition for the acts constituting different types of violence—along with information on their frequency and any associated instructions—hinders replication (Costa & Barros, 2016), as well as comparisons and broader generalizations.

The disparities in the rates of bidirectional IPV across studies may also be due to a lack of agreement between partners’ reports of IPV (Stover et al., 2022). Indeed, most studies included in this systematic review relied solely on reports from one partner, with no assessment of interpartner agreement. Using data from only one member of the couple raises concerns about the validity of the findings and increases the likelihood of social desirability bias (Machado et al., 2024). Only five studies incorporated reports from both partners and included some evaluations of interpartner agreement. This finding aligns with a prior literature review by Armstrong et al. (2002), which noted that five of 15 reviewed studies reported some level of agreement between partners in heterosexual couples. Despite moderate levels of agreement observed in our review, three of the five studies relied on percentage-based agreement indices. While percentages of agreement are straightforward to interpret, they are subject to bias due to the occurrence of behaviors and do not account for chance (Simpson & Christensen, 2005). Furthermore, the extent to which chance contributes to agreement is often unreported (Capinha et al., 2024). To address these limitations, alternative methods such as Kendall’s Tau-b, Intraclass Correlations, Cohen’s Kappa, and Gwet’s AC1 have been proposed (Capinha et al., 2024). However, only two studies in this review used such methods, relying on a single agreement index. Additionally, the use of varied instruments to measure BV (e.g., CTS, specific questions) and differing data collection methods (e.g., web-based surveys, face-to-face interviews, telephone interviews) complicates drawing conclusions about the reliability of one partner’s report compared to both partners’ accounts. These findings may also be influenced by methodological inconsistencies in the instruments and data collection processes. Assessing inter-partner agreement is crucial, as greater consistency in partners’ IPV reports enhances the reliability of IPV scores (Capinha et al., 2024). Using both partners’ perspectives offers deeper insights into individual perceptions and the couple’s dynamics. However, this approach is not always feasible or cost effective. IPV reports are particularly vulnerable to social desirability bias (Moffitt et al., 1997) and may be affected by factors such as memory recall, education, shame, and measurement error, which contribute to discrepancies between partners’ accounts (Armstrong et al., 2002; Moffitt et al., 1997; Yoshikawa et al., 2021). Additionally, individuals may fail to identify themselves as either victims or perpetrators of IPV, further complicating assessments (Straus et al., 1996). Future research should prioritize examining the alignment between one partner’s self-reported IPV perpetration and the other partner’s self-reported victimization across various samples and measures. Employing multiple agreement indices, as findings may vary depending on the method used (Capinha et al., 2024; Yoshikawa et al., 2021). This approach would enhance understanding and improve the reliability of IPV assessments.

Despite the contributions, some limitations should be noted. First, half of the studies were conducted in America, which hinders a comprehensive understanding of the instruments utilized to assess BV in other continents. Second, the data collection was restricted to peer-reviewed studies in English, Spanish, and Portuguese, which may have excluded important perspectives from other languages. Third, the small number of studies examining instruments other than the CTS limits our ability to draw definitive conclusions regarding the differences in BV prevalence across various instruments and the psychometric properties of alternative tools. Fourth, most outcomes were assessed through web-based platforms, underscoring the need for alternative approaches to more effectively capture the complexity of BV, especially in areas such as sexual abuse prevalence. Finally, the methodological heterogeneity among the included studies posed challenges for direct comparisons. While the review included several high-quality studies, the presence of articles with only acceptable quality (scores in the 50% range) highlights the need for improvements in the design and execution of future research.

In conclusion, BV remains a widespread phenomenon within IPV across community, forensic, and clinical samples, representing a significant public health issue that demands an integrated approach combining public health policies with medical, psychological, and social interventions (Rakovec-Felser, 2014). This calls for urgent attention from researchers, policymakers, and practitioners alike. However, progress in both research and intervention efforts is hindered by the lack of comprehensive instruments to accurately measure bidirectional IPV. This systematic review aimed to consolidate and organize existing research on the instruments used to assess bidirectional IPV among adult men and women. Despite certain limitations, our findings suggest that the CTS remains the most commonly used instrument for assessing BV, valued for its strong psychometric properties, irrespective of sample type or data collection method. The second most employed instrument consists of researcher-designed questions based on IPV definitions. Notably, prevalence rates of BV do not appear to vary depending on the instrument used, except in the case of BV rates reported among men. However, the small number of available studies makes it difficult to draw definitive conclusions (see Table 3). Furthermore, the limited number of studies examining interpartner agreement complicates reliable estimations of BV prevalence, as most studies rely solely on self-reports from one partner. This highlights the need for more comprehensive, multi-source data to accurately assess the scope of bidirectional IPV.

**Table 3.** Key Findings of the Systematic Review.

**Key Findings**
Publications on BV only began to emerge in 2012.
CTS was the most utilized instrument to assess bidirectional IPV.
CTS demonstrated robust psychometric properties, proving reliability across diverse sample types and data collection methods.
Prevalence rates for BV show consistency across various instruments, except when based on male reports.

This study provides valuable insights into assessment and intervention practices, as well as the development of public policies in the field of IPV, with a particular focus on bidirectional IPV (see Table 4). The adoption of more comprehensive assessment tools, which include clear instructions and precise operational definitions for each type and form of violence, while accounting for the consequences, motives, and contextual factors, could significantly enhance the identification and management of BV. Future research should focus on identifying instruments with superior psychometric characteristics, sensitivity, and specificity, tailored to diverse populations, contexts, and methodologies (Costa & Barros, 2016). Computer-assisted surveys have shown promise in increasing confidentiality and reducing social desirability bias compared to interviews or paper-and-pencil formats (Hamby, 2014). Third-party reports, such as those from children exposed to IPV, can also offer alternative perspectives, particularly as studies on witnessed violence often reveal gender asymmetries not captured through direct self-reports (Hamby, 2014). Additionally, innovative approaches like vignettes and virtual reality (VR) are gaining attention for their potential to address contextual nuances. Vignettes allow for controlled exploration of how individuals interpret and respond to various IPV scenarios (Hine et al., 2022), while VR enables participants to engage with immersive, realistic environments, providing ecologically valid insights into IPV dynamics (Caixinha et al., submited). It is also critical that assessment instruments for bidirectional IPV incorporate reports from both partners to ensure a more accurate and reliable evaluation. Additionally, future studies should seek to explore more deeply the dynamics of power and control that characterize BV, prioritizing longitudinal approaches that analyze its progression over time, as well as the interplay between contextual and individual factors. Mixed methods, combining both quantitative and qualitative data, are recommended to enrich the understanding of this complex phenomenon. Furthermore, the development of prevention and intervention programs must address the multifaceted nature of BV. These programs should consider the contextual factors that contribute to BV, while promoting strategies for equitable conflict resolution and avoiding the reinforcement of unilateral blame.

**Table 4.** Implications for Research, Practice, and Policy.

**Implications**
It is recommended to use more comprehensive instruments that include clear instructions and precise definitions for each type and form of violence, while also considering the consequences, motives, and contextual factors.
Future research should focus on identifying instruments with improved psychometric properties, as well as better sensitivity and specificity, for diverse populations, contexts, and methodologies.
Longitudinal studies are needed to examine the progression of BV over time and its interaction with contextual and individual factors.
Future research should incorporate mixed methods, combining both quantitative and qualitative data to provide a more comprehensive understanding of BV.
